# Supplementary material for: Common and Differential Dynamics of the Function of Peripheral Blood Mononuclear Cells between Holstein and Jersey Cows in Heat-Stress Environment
Source: Animals (Basel). 2020 Dec 24;11(1):19. doi: 10.3390/ani11010019 (PMC7824059; doi:10.3390/ani11010019)
Supplement: Supplementary file 1 [file animals-11-00019-s001.zip › animals-995477-supplementary/Figure S1 and S2.docx]

**
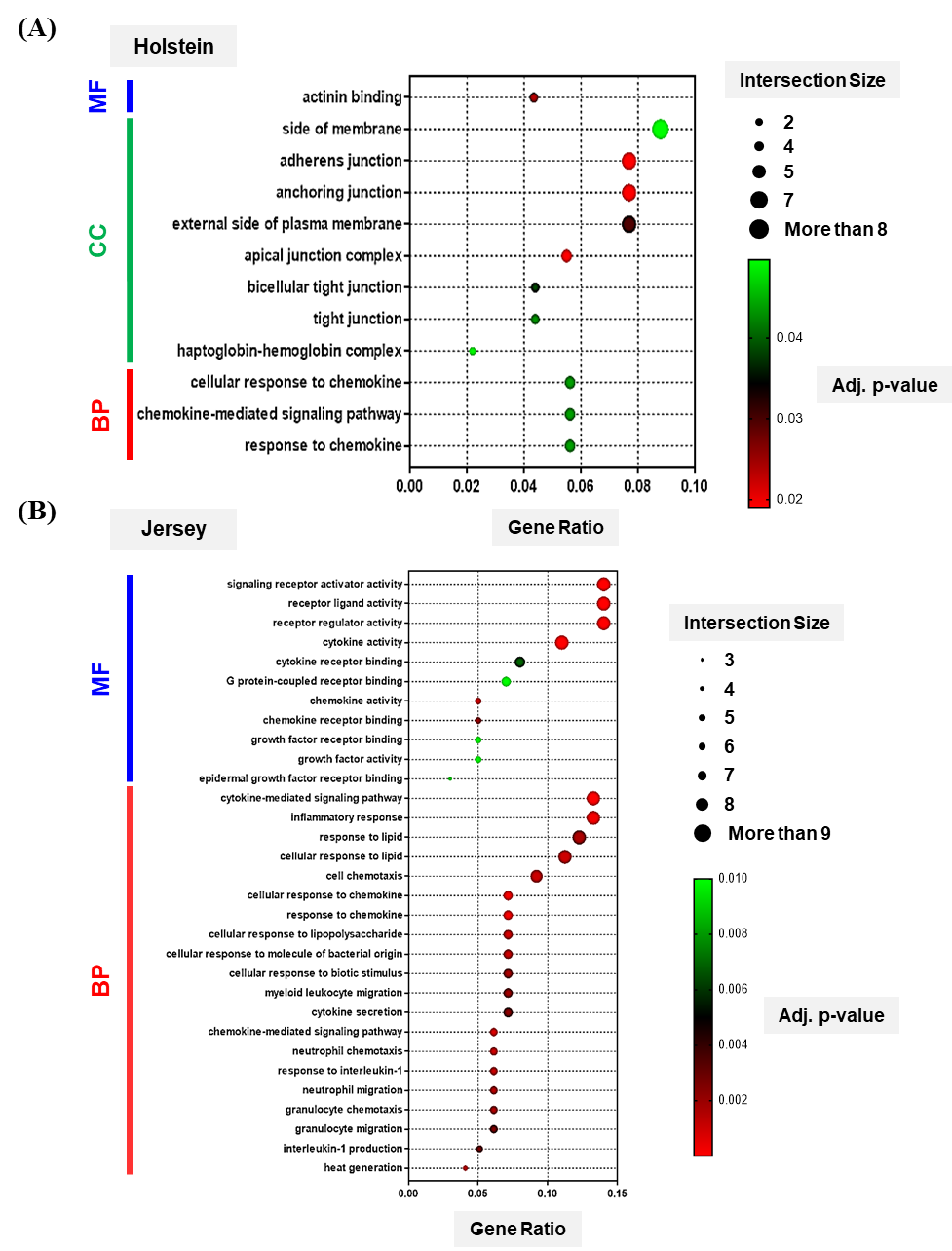
**

**Figure S1.** Gene Ontology terms for differentially expressed genes of two breeds of dairy cows. GO terms for the biological process (BP), cellular component (CC), and molecular function (MF) ontologies up to top 20 ranks for Holstein cows (A) and Jersey cows (B) (*p* < 0.05). Very small or large size terms were filtered out to reduce the exaggeration of statistical significance. After term size filtering, the top 20 terms were expressed as dot plots by adjusted p-values by hypergeometric and multiple testing correction. The intersection size means the number of unique DEGs that are associated with the terms, and the gene ratio means the number of DEGs associated with that term divided by the number of DEGs related to that functional category**.**

**
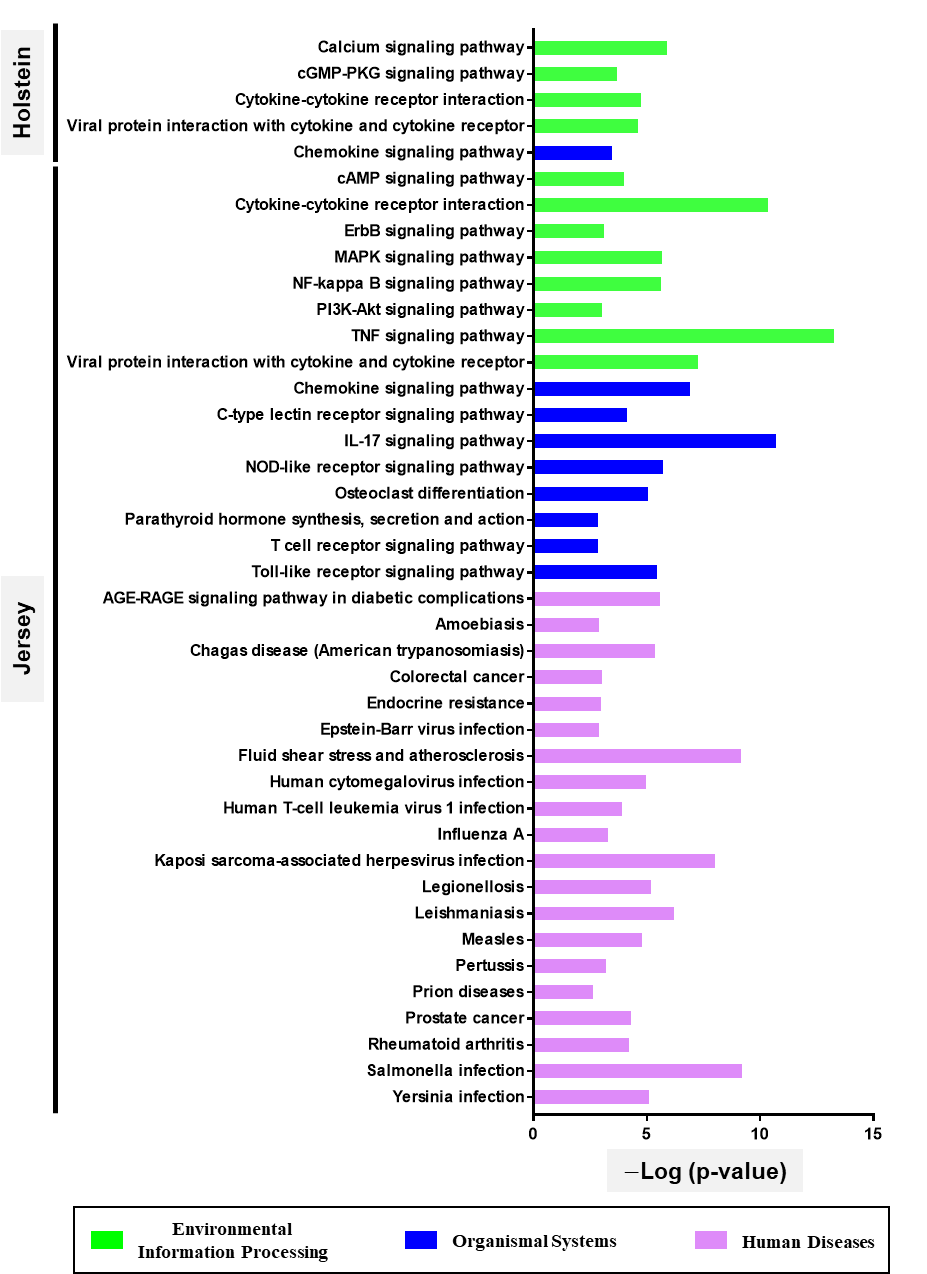
**

**Figure S2.** The cut-off KEGG pathways for two breeds of dairy cows. The significant KEGG pathways were determined by the modified fisher's exact test (*p* < 0.05). Then, the p-values of pathways were corrected by the false discovery rate (FDR) to generate the cut-off KEGG pathways (FDR<0.01). The cut-off KEGG pathways were represented by 3 categories (environmental information processing, organismal systems, human diseases) and transformed to log value.
