# Supplementary material for: Common and Differential Dynamics of the Function of Peripheral Blood Mononuclear Cells between Holstein and Jersey Cows in Heat-Stress Environment
Source: Animals (Basel). 2020 Dec 24;11(1):19. doi: 10.3390/ani11010019 (PMC7824059; doi:10.3390/ani11010019)
Supplement: Supplementary file 1 [file animals-11-00019-s001.zip › animals-995477-supplementary/Table S1 and S2.docx]

**Table S1.** Ingredients and nutrients of the experimental diets in this study

| **Item** | **Amount** |
| --- | --- |
| Ingredients composition, % of DM |  |
| Concentrate | 15.3 |
| Soybean meal | 2.4 |
| Corn silage | 47.2 |
| Alfalfa hay | 7.1 |
| Tall fescue | 9.4 |
| Timothy | 5.9 |
| Energy booster^1^ | 7.1 |
| Cash Gold^1^ | 4.5 |
| Lyzin-Plus^2^ | 0.2 |
| Limestone | 0.2 |
| Zin Care^1^ | 0.1 |
| Supex-F^1^ | 0.5 |
| Trace minerals^3^ | 0.05 |
| Vitamins premix^4^ | 0.05 |
| Chemical composition |  |
| Dry matter (DM), % | 53.2 |
| Crude protein, % of DM | 10 |
| Neutral detergent fiber, % of DM | 28.2 |
| Acid detergent fiber, % of DM | 16.9 |
| Calcium, % of DM | 0.4 |
| Phosphorus, % of DM | 0.15 |

^1^Cofavet, Cheonan, Republic of Korea. ^2^A.N.Tech, Cheonan, Republic of Korea. ^3^Contained 0.40% Mg, 0.20% K, 4.00% S, 0.08% Na, 0.03% Cl, 400 mg of Fe/kg, 60,042 mg of Zn/kg, 16,125 mg of Cu/kg, 42,375 mg of Mn/kg. ^4^Provided approximately 5,000 KIU of vitamin A/kg, 1,000 KIU of vitamin D/kg, 33,500 mg of vitamin E/kg, and 2,400 mg of vitamin C/kg.

**Table S2.** Summary sequencing statistics for dairy cow PBMC samples

|  | **ID** | **Total read bases** | **Total reads** | **Mapped reads (%)** | **GC (%)** | **Q20 (%)** | **Q30 (%)** |
| --- | --- | --- | --- | --- | --- | --- | --- |
| **Holstein** | M_H1 | 6,732,401,407 | 66,976,236 | 65,634,803 (98.0%) | 44.32 | 98.64 | 95.20 |
|  | M_H2 | 6,392,720,753 | 63,652,100 | 60,892,137 (95.66%) | 46.14 | 98.40 | 94.54 |
|  | M_H3 | 5,451,526,099 | 54,217,730 | 51,294,878 (94.61%) | 46.00 | 98.83 | 95.77 |
|  | M_H4 | 5,250,248,487 | 52,222,780 | 50,105,522 (95.95%) | 45.94 | 98.72 | 95.46 |
|  | M_H5 | 5,417,393,994 | 53,909,406 | 51,403,698 (95.35%) | 45.71 | 98.73 | 95.47 |
|  | A_H1 | 5,285,767,384 | 52,578,416 | 51,028,069 (97.05%) | 45.52 | 98.79 | 95.63 |
|  | A_H2 | 5,837,676,662 | 58,068,022 | 55,734,625 (95.98%) | 46.01 | 98.81 | 95.70 |
|  | A_H3 | 6,846,064,255 | 68,105,894 | 66,065,210 (97.0%) | 44.8 | 98.72 | 95.43 |
|  | A_H4 | 5,088,793,286 | 50,609,822 | 49,195,222 (97.2%) | 43.9 | 98.79 | 95.62 |
|  | A_H5 | 7,027,026,148 | 69,915,058 | 65,789,114 (94.1%) | 44.99 | 98.74 | 95.51 |
| **Jersey** | M_J1 | 6,791,305,770 | 67,526,766 | 65,275,308 (96.67%) | 45.54 | 98.81 | 95.69 |
|  | M_J4 | 4,921,738,691 | 48,997,768 | 43,179,237 (88.12%) | 49.93 | 98.67 | 95.40 |
|  | M_J5 | 5,332,022,949 | 53,070,942 | 51,097,761 (96.28%) | 45.31 | 98.66 | 95.26 |
|  | M_J6 | 5,697,714,745 | 56,688,608 | 53,798,924 (94.9%) | 45.87 | 98.78 | 95.63 |
|  | A_J1 | 6,843,176,905 | 68,074,168 | 66,407,329 (97.55%) | 44.59 | 98.76 | 95.54 |
|  | A_J2 | 5,229,401,965 | 52,018,654 | 50,526,859 (97.13%) | 44.52 | 98.77 | 95.59 |
|  | A_J4 | 7,080,113,118 | 70,464,696 | 63,247,938 (89.76%) | 46.99 | 98.66 | 95.29 |
|  | A_J5 | 5,289,778,783 | 52,621,316 | 51,174,880 (97.25%) | 44.96 | 98.79 | 95.66 |
|  | A_J6 | 6,011,898,426 | 59,825,986 | 58,136,650 (97.18%) | 45.21 | 98.65 | 95.29 |
